# CD4+ T Cell Depletion, Immune Activation and Increased Production of Regulatory T Cells in the Thymus of HIV-Infected Individuals

**DOI:** 10.1371/journal.pone.0010788

**Published:** 2010-05-24

**Authors:** Alessandra Bandera, Giulio Ferrario, Marina Saresella, Ivana Marventano, Alessandro Soria, Fabio Zanini, Francesca Sabbatini, Monica Airoldi, Giulia Marchetti, Fabio Franzetti, Daria Trabattoni, Mario Clerici, Andrea Gori

**Affiliations:** 1 Division of Infectious Diseases, Department of Internal Medicine, San Gerardo Hospital, University of Milan-Bicocca, Monza, Italy; 2 Infectious Diseases Section, Department of Clinical Sciences, Luigi Sacco Hospital, University of Milan, Milan, Italy; 3 Don C. Gnocchi Foundation IRCCS, Milan, Italy; 4 Clinic of Infectious Diseases, San Paolo Hospital, University of Milan, Milan, Italy; 5 Chair of Immunology, Department of Preclinical Sciences, LITA VIALBA, University of Milan, Milan, Italy; 6 Chair of Immunology, Department of Biomedical Sciences and Technologies, University of Milan, Milan, Italy; University of California San Francisco, United States of America

## Abstract

Mechanisms by which HIV affects the thymus are multiple and only partially known, and the role of thymic dysfunction in HIV/AIDS immunopathogenesis remains poorly understood. To evaluate the effects of HIV infection on intra-thymic precursors of T cells in HIV-infected adults, we conducted a detailed immunophenotypic study of thymic tissue isolated from 7 HIV-infected and 10 HIV-negative adults who were to undergo heart surgery. We found that thymuses of HIV-infected individuals were characterized by a relative depletion of CD4+ single positive T cells and a corresponding enrichment of CD8+ single positive T cells. In addition, thymocytes derived from HIV-infected subjects showed increased levels of activated and proliferating cells. Our analysis also revealed a decreased expression of interleukin-7 receptor in early thymocytes from HIV-infected individuals, along with an increase in this same expression in mature double- and single-positive cells. Frequency of regulatory T cells (CD25+FoxP3+) was significantly increased in HIV-infected thymuses, particularly in priorly-committed CD4 single positive cells. Our data suggest that HIV infection is associated with a complex set of changes in the immunophenotype of thymocytes, including a reduction of intrathymic CD4+ T cell precursors, increased expression of activation markers, changes in the expression pattern of IL-7R and enrichment of T regulatory cells generation.

## Introduction

HIV infection initiates a series of complex events culminating in profound immunosuppression caused by functional abnormalities and quantitative depletion of CD4+ T lymphocytes. The mechanism(s) responsible for the progressive CD4 cell count decline seen in untreated HIV infection remain a matter of controversy [1]–[4]. Current observations suggest that direct infection of target cells is only partially responsible for T-cell depletion. A more complex model which also includes alterations in immune activation, T-cell turnover and homeostatic regulation, is now favoured [5]–[8].

HIV infection leads to sustained immune activation and to major alterations in T cell homeostasis [9]–[13]. In particular, *naive* cells, CD4 and CD8 alike, are progressively depleted, possibly as a consequence of their frequent activation and differentiation into memory cells caused by chronic and high antigenic stimulation.

The thymus is the primary organ of thymopoiesis and is highly active during early life. While thymic function may not be necessary after puberty in most individuals, significant thymocyte and T cell depletion can occur as a result of chemotherapy, bone marrow transplant or HIV infection. In each of these three scenarios, the thymus is required to play a significant role in achieving complete immune recovery [14]–[17].

Impairment of thymic T-cell production in AIDS pathogenesis was originally proposed following studies which demonstrated destruction of thymic structure, lack of thymocytes and infiltration of activated cells in thymuses of AIDS patients [18]–[19]. Measurement of TCR rearrangement excision circles (TREC), used to assess thymic output in individuals with HIV infection, has failed to produce clear conclusions. Douek and collaborators found decreased T cell TREC content and reduced proportions of *naïve* T cells during early HIV infection, resulting from a combination of increased T cell proliferation and decreased thymic output [20]. It is not possible to distinguish the true effects of peripheral events such as T cell activation and expansion on CD4+ TREC content, either during HIV infection or during antiretroviral induced immune-reconstitution. For this reason, caution in interpreting TREC assay is required [21]. Dion and coworkers studied an alternate sj/bTREC ratio which directly reflects precursors cell proliferation in the thymus, thereby avoiding confusion caused by peripheral T cell division. They revealed that HIV infection disrupts the development of T cells early in the course of disease progression, and that this thymic defect is partly compensated in the periphery during the same period. Increased DβJβ TREC frequencies point to a reduced role of the virus in cell death, with a major part being played instead by cytokine-mediated inhibition [22]. Studies on SIV-infected rhesus macaques revealed minimal impact from thymectomy on the peripheral T-cell compartment [Picker, unpublished data], despite a lack of an extrathymic source of naïve T cells [23].

Our *ex-vivo* study was aimed at an in-depth analysis of the effect of HIV infection on intra-thymic precursor T cells. In particular, thymic tissue of HIV-infected patients were phenotypically studied to evaluate the effect of HIV infection on thymic precursors of CD4+ T cells. The potential role of proliferation and immune activation was also considered by evaluating the impact of peripheral immune activation on thymocytes. Given IL-7's essential role in early human T-cell development and homeostasis [24]–[26], we considered it important to evaluate the expression of IL-7 receptor on thymic cells of both HIV-infected and uninfected subjects. Moreover, since regulatory T cells (Treg) develop in the thymus [27] and are critical for the control of immune responses [28], we analyzed the impact of HIV on the development of CD4+CD25+ T cells in the thymus.

## Methods

### Study design

We performed a cross-sectional, observational, institutional review board-approved study (Institute of Infectious Diseases, Luigi Sacco Hospital, Milan) of HIV-infected and HIV-negative adult donors undergoing cardiac surgery for coronary disease or cardiac valve disease at Luigi Sacco Hospital, University of Milan.

Seven HIV-infected and 10 age-matched HIV-negative adult donors were enrolled at the Institute of Infectious and Tropical Diseases, Luigi Sacco Hospital, University of Milan (table 1). Thymic tissues were collected during cardiac surgery in adult patients. Patients participating in this study gave written informed consent according to the Declaration of Helsinki. Adult donors with autoimmune diseases or donors treated with immunosuppressive drugs were excluded.

**Table 1:** HIV-infected subjects characteristics at thymectomy.

Patient	Age (years)	Sex	CDC stage	CD4+ T cells (cells/µl)	Plasma HIV-RNA log10 (cp/ml)	ARV therapy^a^
1	32	M	A2	618	4.89	none
2	41	M	A1	509	5.02	none
3	51	F	A3	763	1.69	ddC+AZT
4	48	M	A2	481	4.59	none
5	43	M	C3	328	1.69	3TC+DRV/r+RAL
6	42	F	B3	40	4.20	none
7	53	M	C3	379	1.69	FTC+TDF+DRV/r+RAL

a current antiretroviral therapy.

### Tissue collection and cell preparation

Immediately after surgery, thymus tissue was separated from the fibrotic capsule and from visible blood-vessels, mechanically disaggregated using a scalpel and scissors followed by pipetting several times with phosphate buffered saline (PBS). A single-cell suspension from the thymus was obtained by gently passing the collected PBS and tissue fragments through a sterile 70 mm stainless-steel sieve. Cell suspensions were centrifuged and fatty debris was discarded.

Freshly isolated cells from each sample were filtered through a 50 mm sterile nylon mesh and examined by means of flow cytometry.

All steps were performed in asepsis using sterile PBS containing 0.1% bovine serum albumin.

### Flow cytometry

Flow cytometry was performed using a FACScan flow cytometer (FC500 Beckman Coulter Miami, FL). Cells were analysed immediately after immunostaining using forward-scatter and side-scatter signals to establish the thymocyte gate and to exclude dead cells, debris and cell clumps. Fluorescence signals were collected in log mode. A minimum of 50.000 cells of interest were acquired for each sample and data were analysed using CXP analysis software. Immunostaining was performed using conjugated monoclonal antibodies (mAbs) to: CD1a, CD3, CD4, CD8, CD27, CD34, CD69, CD127 (IL-7 receptor a-chain), CCR5, CXCR4, Ki67, TCRαβ and TCRγδ, CD25, FoxP3 (Becton Dickinson Biosciences, San Jose, CA and Beckman Coulter, Miami, FL). The mAbs were conjugated with fluorescent dyes fluorescein-isothiocyanate (FITC), phycoerythrin (PE), phycoerythrin-texas-red (ECD), phycoerythrin-cyanin-7 (PC7), phycoerythrin-cyanin-5 (PC5) and appropriately combined to assess the thymic cell subset of interest in five-colour fluorescence assays. Irrelevant isotypic mAbs were used as negative control.

**Figure 1:**
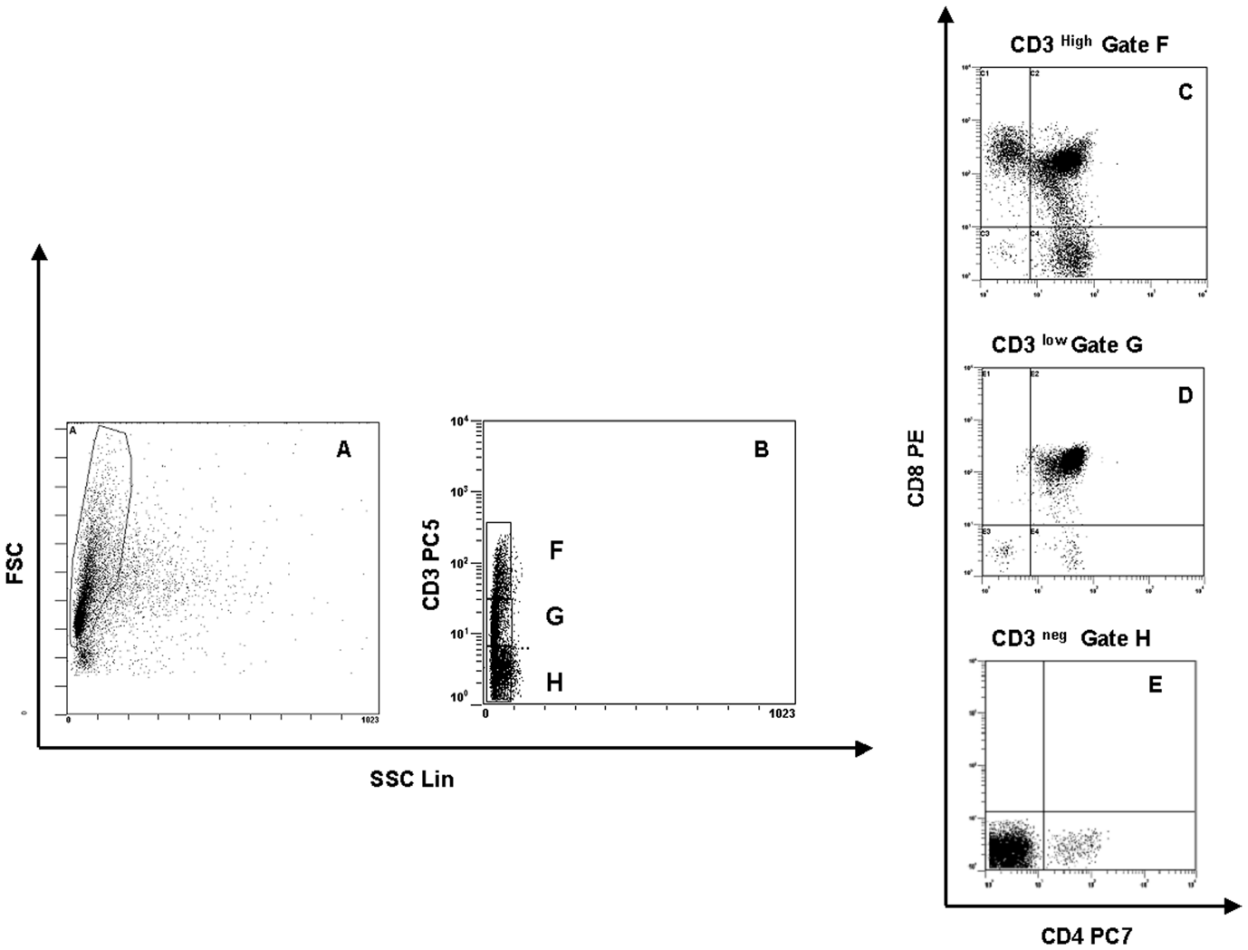
Flow cytometry analysis of thymocytes. Thymocytes were characterized by their forward (FSC) and side scattering (SSC) properties(A). Thymocytes were gated by their CD3+ expression and SSC characteristics (B) in CD3 **^High^**(gate F), CD3 **^low^** (gate G), CD3 **^neg^** (gate H). Dot plots CD3 **^High^** DP and SP (C) CD3 **^low^** DP (D) and CD3 **^neg^** TN and ISP (E) thymocytes are showed. DP = double positive; TN = triple negative; SP = single positive; ISP:immature single positive (CD4+).

Different phenotypes were used to evaluate thymopoiesis: CD34+CD1a-; CD34+CD1a+; CD3-CD4-CD8- (triple negative, TN); CD3-CD4+CD8- (immature single positive CD4+, ISP4+); CD3-CD4+CD8+ (CD3- double positive,CD3-DP); CD3+CD4+CD8+ (CD3+ double positive, CD3+DP); CD3+CD4+CD8- (single positive, SP CD4+); CD3+CD4-CD8+ (single positive, SP CD8+) (figure 1).

### Statistical analysis

Comparisons between HIV-positive and HIV-negative patients were made using a Mann-Whitney U-test. Correlation analysis was made using a non-parametric test. Statistical analysis was performed using the SAS statistical package.

## Results

### Flow cytometry evaluation of thymopoietic stages

**Figure 2:**
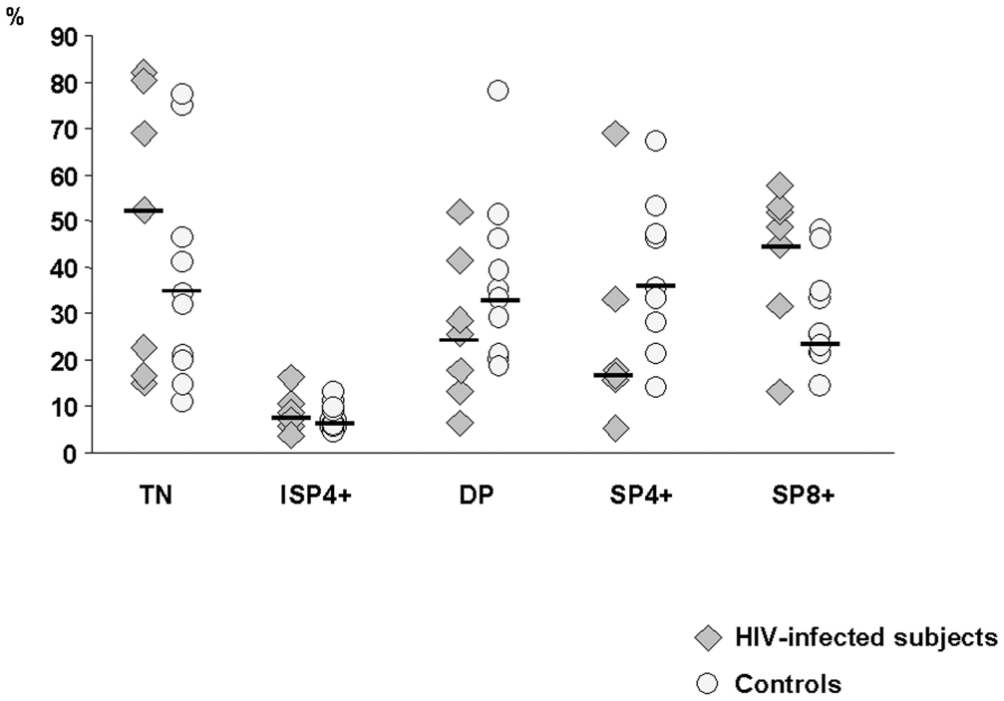
Flow cytometry evaluation of thymopoietic stages in HIV-infected (grey triangles) and uninfected controls (white circles). All thymopoietic stages were detected in both HIV+ and HIV- thymic tissues, but these stages were skewed in HIV-infected compared to uninfected thymuses. Thus, whereas the percentage of immature triple negative cells (CD3-CD4-CD8-, TN) and of immature single positive CD4+ cells (ISP4+) was comparable in HIV+ and in HIV- patients, an increase of CD3+CD4-CD8+ single positive (SP8+) cells (46.0% versus 22.5%) as well as a reduction of double-positive (DP) (CD3brightCD4+CD8+: 23.0% versus 31.05%) and CD3+CD4+CD8- single positive (SP4+) cells (13.9% versus 37.7%) were observed in HIV+ compared to HIV- subjects.

Phenotypic subpopulation analysis was performed on input cells using multiparameter flow cytometry. Percentages of CD34+CD1a- and CD34+CD1a+ thymocytes were similar in the two groups and represented the smallest proportions of thymocytes, where CD34+CD1a- were 0.04-2.5% of cells in the HIV-infected group and 0.02–4% in uninfected controls and CD34+CD1a+ were 0.1–0.13% of cells in HIV-infected thymuses and 0.1–1.3% in uninfected controls. Percentages of triple negative and immature single positive CD4+ cells (CD3-CD4+CD8- cells) did not significantly differ in HIV-infected and HIV-negative subjects (TN: HIV+ 49.8%; HIV- 29%; ISP4+: HIV+ 4.6%; HIV- 4.15%), indicating a normal presence of intrathymic precursors in HIV-infected thymuses (figure 2).

T cells actively undergoing thymopoiesis show moderate CD3 expression. Bright CD3 expression is a hallmark of mature T cells which are either preparing to emigrate or are residing in the perivascular space of the thymus. Frequency of CD3-low cells in HIV-infected individuals was 71%; this result was similar to that observed in HIV-negative subjects (67%), confirming the presence of active thymopoiesis in thymic tissue of both groups.

Another measure of thymopoiesis is frequency of CD4+CD8+ double-positive T cells. Compared to HIV-uninfected individuals, HIV-infected subjects had lower levels of CD4+CD8+ double-positive cells (CD3-low DP HIV+ 33.5%; HIV- 50.5%; CD3-bright DP HIV+ 23%; HIV- 31.05%).

In evaluating single positive cells, we found lower expressions of CD4+ single positive cells in HIV-infected patients in comparison to HIV-negative subjects (HIV+ 13.9%; HIV- 37.7%). Conversely, HIV+ subjects displayed gher levels of CD8+ single positive cells than uninfected controls (HIV+ 46%; HIV- 22.5%).

To characterize the imbalance between CD4+ SP and CD8+ SP T cells in the two groups of subjects, we calculated the CD4+ SP/CD8+ SP ratio. The comparison of HIV-infected with healthy thymuses controls showed significantly lower levels of CD4+ SP/CD8+ SP T cells in the HIV-infected group compared to controls (HIV+ 0.30, HIV-1.44, p = 0.03), thus confirming the relative depletion of CD4+ single positive T cells and the corresponding enrichment of CD8+ single positive T cells in HIV-infected thymuses.

Taken together, these results indicate that there was active thymopoiesis in thymic tissues isolated from HIV-infected individuals. However, thymic processes in HIV-infected subjects appear to be characterized by a less-extensive expression of CD4-positive cells and a compensative bias toward a CD8-positive pattern.

### Immune activation and proliferation of thymocytes

**Figure 3:**
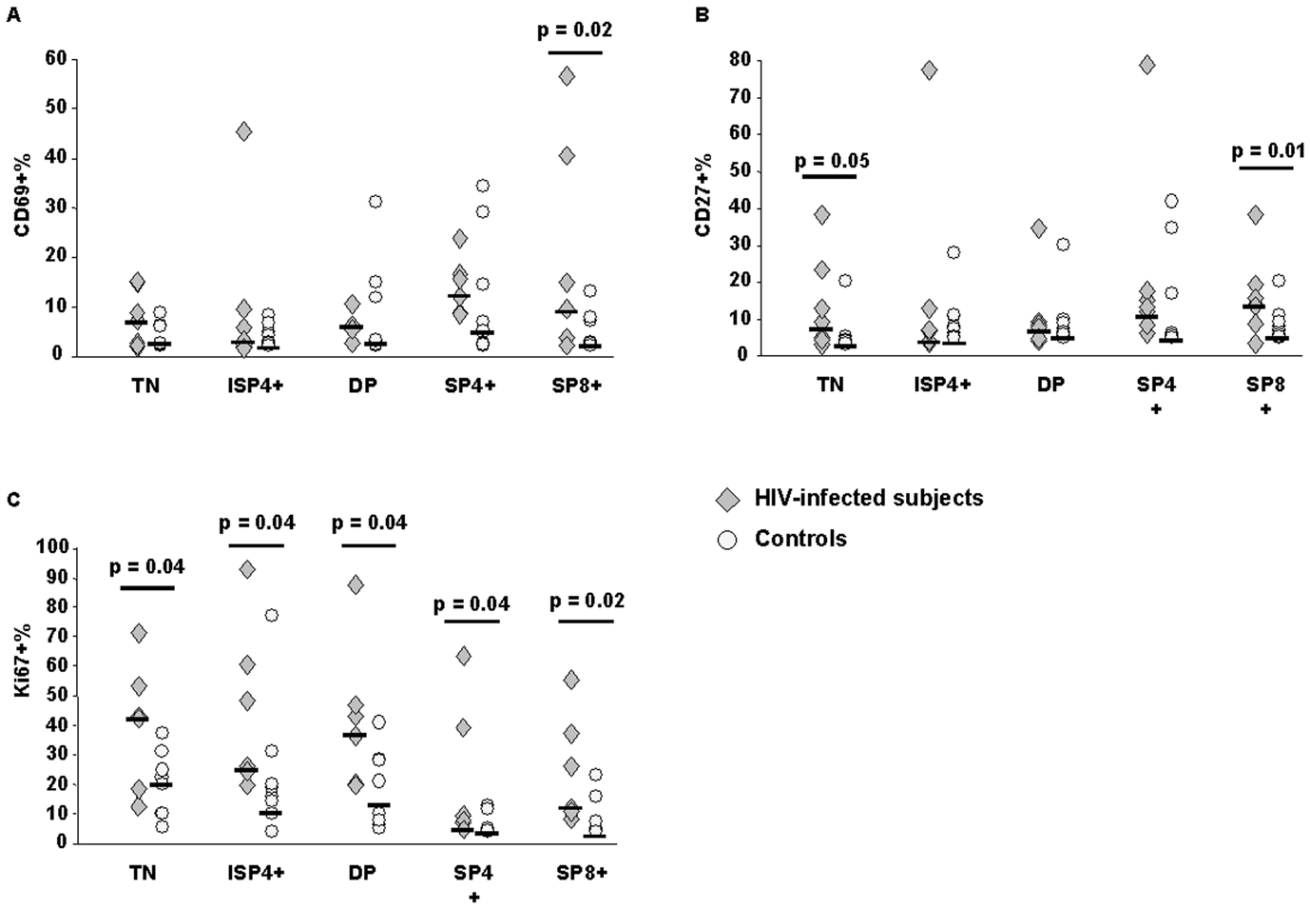
Expression of CD69, CD27 and Ki67 on thymocytes in HIV-infected (grey triangles) and uninfected controls (white circles). HIV-infected subjects showed significantly higher percentages of CD69(a) expression in single positive CD8+ cells (HIV+ 7.9%; HIV- 0.6%; p = 0.02) compared to HIV-uninfected controls. A similar trend toward higher levels of CD27 (b) expression was seen in HIV-infected patients compared to HIV-negative subjects. This was observed in triple negative cells (HIV+ 6.0%; HIV- 0.625%, p = 0.05), in CD3+ double positive cells (HIV+ 4.7%; HIV- 1.25%), and in single positive cells (SP4+ HIV+ 10.2%, HIV- 1.0%; SP8+ HIV+11.0%, HIV-1.6%, p = 0.03). Significantly higher levels of Ki67 thymocytes (c) were detected in triple negative cells (HIV+ 39.5%, HIV- 17.2%, p = 0.04) and in double positive and CD8+ single positive cells of HIV-infected patients compared to uninfected controls (CD3+DP HIV+ 36.35%, HIV-11.45%, p = 0.04; SP4+ HIV+ 5.65%, HIV- 0.45%, p = 0.04; SP8+ HIV+ 15.9%, HIV- 1.15%, p = 0.02).

Immune activation was measured by expression of CD27 [29] and CD69 on thymocytes (figure 3). A trend toward higher levels of CD27 expression was seen in HIV-infected individuals compared to HIV-negative subjects. This was observed both in triple negative cells (HIV+ 6%; HIV- 0.625%, p = 0.05), in CD3+ double positive cells (HIV+ 4.7%; HIV- 1.25%) and in single positive cells (SP4+ HIV+ 10.2%, HIV- 1%; SP8+ HIV+11%, HIV-1.6%, p = 0.03). A similar trend was observed when expression of CD69 was analyzed. HIV-infected subjects had higher percentages of CD69 expression in TN thymocytes (HIV+ 5.5%, HIV- 0.5%) and in single positive CD8+ cells (HIV+ 7.9%; HIV- 0.6%; p = 0.02) compared to HIV-uninfected controls.

Our results showed that, despite an apparently similar rate of thymopoiesis, thymic tissues of HIV-infected subjects are characterized by thymocyte immune hyperactivation involving either mature T cells or T cells actively undergoing thymopoiesis.

**Figure 4:**
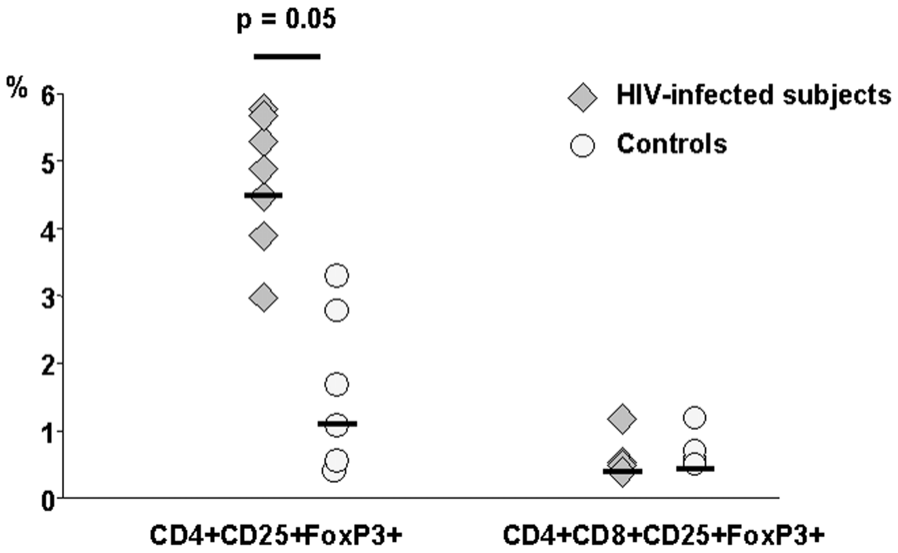
Expression of CD25 and FoxP3 on CD4+ SP and CD3+DP thymocytes in HIV-infected (grey triangles) and uninfected controls (white circles). The expression of CD25 and FoxP3 on CD4+ SP cells was significantly increased in thymuses of HIV-infected individuals compared to uninfected controls (HIV+ 4.3%, HIV- 1.3%, p = 0.05), whereas the expression of CD25 and FoxP3 on CD4+CD8+ DP cells was comparable in the two groups of subjects (HIV+ 0.22%, HIV- 0.19%).

*Ex-vivo* staining of thymocyte subsets with Ki67 antibodies confirmed previous data showing the presence of extensive thymocyte proliferation in both HIV-infected and HIV–uninfected thymuses. However, significantly higher levels of Ki67 thymocytes were detected in triple negative cells (HIV+ 39.5%, HIV- 17.2%, p = 0.04) of HIV-infected subjects and at each subsequent stage of thymopoiesis (CD3-DP HIV+ 59.5%, HIV- 20.55%, p = 0.04; CD3+DP HIV+ 36.35%, HIV-11.45%, p = 0.04; SP4+ HIV+ 5.65%, HIV- 0.45%, p = 0.04; SP8+ HIV+ 15.9%, HIV- 1.15%, p = 0.02) (figure 4A).

Overall, our data show that, as opposed to HIV- controls, T-cell activation and proliferation is higher in HIV-infected thymuses and involves both early-stage thymocytes and mature thymocytes.

### Interleukin-7 (IL-7) receptor expression on thymocytes

IL-7 signalling via the IL-7 receptor plays a critical role in thymocyte development. Because of this, CD127 expression on different thymocyte subsets was deemed to be important and therefore evaluated. Interestingly, HIV-infected subjects displayed lower levels of CD127 expression on triple negative cells (HIV+ 0.5%, HIV- 2.95%), thus suggesting a possible impairment of IL-7 signalling in HIV-infected thymocytes during the earlier intrathymic maturation stages or, otherwise, an increase binding of IL-7. However, expression of CD127 on later thymopoietic stages was higher in HIV-infected individuals compared to results obtained in HIV-negative controls (CD3+DP HIV+ 2.3% HIV- 1.3%; SPCD4+ HIV+ 5.8%, HIV-4.6%), suggesting a maintenance of an IL-7 compensatory feedback in HIV infection.

### Expression of chemokine receptors on thymocytes

Thymocytes were stained with CCR5- and CXCR4- specific MAb. In HIV-infected and HIV-uninfected thymuses, CXCR4 was expressed on the majority of thymocytes both in immature stages and in more mature cells. However, analysis of CCR5 expression revealed higher levels of CCR5 on CD3+DP (HIV+ 2.2%, HIV- 0.7%) and SPCD8+ thymocytes (HIV+ 4.71%, HIV-1.7%) in HIV-infected thymus compared to uninfected controls, suggesting a possible role by higher immune activation status in the induction of a more consistent expression of CCR5 on thymocytes.

### Alpha/beta and gamma/delta TCR expression on thymocytes

Differentiation of precursors into mature T cells involves a series of lineage switch-points. These include committing or not committing to the T-cell fate; differentiating or not differentiating into αβ or γδ lineage T cells; and, for an αβ T cell, becoming either a helper (CD4+) or a cytotoxic (CD8+) T cell.

Expression of αβ TCR was significantly higher in CD3+DN thymocytes of HIV+ individuals compared to HIV-negative controls (HIV+ 27.5%, HIV-2.95%, p = 0.02).

γδ TCR was expressed on a minority of thymocytes in both groups of subjects and no differences were observed for various stages of thymopoiesis in HIV-infected compared to HIV–uninfected subjects, suggesting minimal impact from HIV on this thymocyte population, despite its known reputation as a potential target of HIV infection.

### Regulatory T cells

Thymocytes obtained from HIV-infected and uninfected controls were cell surface stained with antibodies to CD25, CD3, CD4 and CD8 prior to intracellular staining of FoxP3 to identify Treg subpopulation.

Expression of CD25 and FoxP3 on CD4+ SP cells was significantly increased in thymuses of HIV-infected individuals compared to uninfected controls (HIV+ 4.3%, HIV- 1.3%, p = 0.05), while the expression of CD25 and FoxP3 on CD4+CD8+ DP cells was comparable (HIV+ 0.22%, HIV- 0.19%) (figure 4B).

These data demonstrate that HIV induces an increase in Treg frequency, particularly in committed CD4 single positive cells. Possibly, this is a result of enhanced Treg survival and function caused by direct infection. Alternatively, it could be connected to bystander effect, mediated by host-derived pro-inflammatory molecules.

### Associations between thymocyte activation, presence of CD4+ and CD8+ single positive thymocytes, CCR5 expression and peripheral CD4+ cell count in HIV-infected patients

To evaluate if the higher levels of thymocyte activation and proliferation found in HIV-infected individuals may result in higher susceptibility to viral infection and exhaustion of regenerative capacity, we investigated if in HIV-infected individuals the fraction of activated/proliferating thymocytes correlate with (i) the lower fraction of CD4+SP and the lower CD4+SP/CD8+SP ratio in the thymus, (ii) increased fraction of thymocytes expressing CCR5, and (iii) CD4+ T-cell count in blood.

In HIV-infected patients, a significant inverse correlation was seen between the expression of Ki67 on triple negative cells and CD4 single positive thymocyte proportion (r = −0.98, p<0.01), thus confirming the association between thymocyte activation and exhaustion of regenerative capacity. Activation of double-positive and CD4 single positive cells, represented by CD69 expression, was associated with peripheral CD4 T cell count (CD69+DP r = 0.85, p = 0.01; CD69+SP4+ r = 0.82, p = 0.02). The expression of CD27 on SP thymocytes was positively associated with CCR5 expression, reaching statistical significance for CD8+ SP cells (r = 0.87, p = 0.05).

## Discussion

We investigated thymopoiesis in HIV-1 infected and healthy adults by performing *ex-vivo* analyses on thymuses obtained from either HIV-infected or HIV-uninfected individuals. Our data indicate that HIV-infection does not impair thymopoiesis per se, but rather induces increased proliferation and activation of thymocytes which may, in the long term, cause clonal exhaustion of T cells and inflammatory damage to lymphoid tissue.

There is considerable evidence indicating that HIV can infect the thymus, both *in vitro* and *in vivo*, compromising the integrity of this immune organ [30]–[32]. Studies of CD4+ T cell depletion and reconstitution in HIV infection have delineated a multifactoral model of immune homeostasis in which composition of *naïve* and *memory*/*effector* pools in blood and lymphoid tissue, thymic function and effects of clinical stage of the disease play a significant role [33]. Current observations have led to a general consensus that CD4 and CD8 T cell activation and turnover are heightened in HIV infection, that HIV infection leads to increased death of CD4+ T cells, that there is a defect in the renewal/replacement mechanisms for CD4+ T cells and that these replacement mechanisms have both peripheral and thymic components.

By studying thymic cells' phenotype of HIV-infected patients and comparing them to healthy controls, we reasoned that if HIV infection induced thymic alterations either through direct lysis or through bystander action, we would be able to detect this effect by analyzing markers of T cell differentiation and activation.

Measures of thymopoiesis indicate the presence of active thymopoiesis in thymic tissues isolated from HIV-infected patients, confirming previous evidence that the thymuses of HIV-positive adult patients can engage in thymopoiesis even after prolonged periods of CD4+ T lymphopenia [34]–[38]. Although immature intrathymic precursors were equally represented in HIV-infected and uninfected patients, HIV-infected thymuses showed less-extensive expression of mature CD4-positive cells and a compensative bias toward a CD8-positive pattern. Possibly, these data represent an indirect marker of CD4-expressing thymocyte destruction by HIV. They also support the role of the thymic component in reducing input of *naïve* CD4 T cells into the peripheral *naïve* T cell pool.

A clear result emerging from this study is the significantly higher proportion of activated and proliferating thymocytes, at all stages of thymopoiesis, in HIV-infected patients compared to controls. Recent discoveries have produced a general consensus that chronic systemic immune activation is a pathognomic feature of progressive HIV infection, detrimental to HIV-infected persons [9]–[11]. It is one of the strongest predictors of disease progression, associated with impaired immune reconstitution in patients on ART. It is also a critical factor which distinguishes pathogenic from non-pathogenic simian immunodeficiency virus infection in nonhuman primates. Significantly, the consequences of immune activation in HIV infection may go far beyond the simple loss of virus-specific CD8+ T cells and bring about a global decline of immune resources. In particular, at the thymic site, immune activation likely caused by bystander mechanisms and sustained by homeostatic proliferation, can produce at least two crucial consequences: generation of activated T cell viral targets, which drives additional viral replication, and inflammatory damage, which may induce long-term thymic dysfunction.

Our data also indicate dichotomous behaviour of the IL-7/IL-7R circuit in HIV-infected thymic tissue. IL-7R expression was found to be lower in early intrathymic maturation stage lymphocytes (CD34+CD3-CD4-CD8-) from HIV-infected patients, a thymic subpopulation previously identified as one of the most responsive to the lymphopoietic signal of IL-7 [26]. Possibly this reflects a dysfunction in IL-7Rα modulation positively correlated to specific surrogate markers of disease progression. Nonetheless, increased expression of IL-7R in both DP and SP cells from HIV-infected thymus could be a consequence of higher IL-7 peripheral concentration, suggesting maintenance of effective IL-7 compensatory feed-back loop.

In order to better understand the mechanism of R5 and X4 HIV-pathogenesis in the human thymus, we sought to quantify the extent of CCR5 and CXCR4 expression on HIV infected human thymocytes. Through phenotypic approach, we discovered that freshly isolated thymocytes from HIV-infected patients have significantly higher expressions of CXCR4 than of CCR5, confirming previous observations about HIV-uninfected human thymus [39]. However, HIV-infected thymus cells also displayed a consistent trend toward higher levels of CCR5 in mature thymocytes compared to uninfected controls. This suggests that higher immune activation status plays an important role in the inducement of consistent expression of CCR5 on thymocytes.

Interestingly, our data, revealing predominantly CXCR4 expression on thymocytes obtained from HIV-infected patients, fail to explain previous studies which found that R5-HIV-1 clones are capable of depleting the vast majority of CD4+ thymocytes from infected SCID-hu thymus-liver grafts [40]. Rather, our data support the hypothesis of a central role of the thymus in the emergence of CXCR4-using quasispecies.

HIV induces abnormal development of Treg in the thymus, by direct infection or by enhancement of their survival and function, mediated by host-derived pro-inflammatory molecules. This enrichment in Treg parallels a similar trend in lymphoid tissue. Previous studies, in fact, found that chronic HIV infection changes Treg cell tissue distribution by increasing CD4+CD25+ in peripheral lymph nodes and mucosal lymphoid tissues, the sites where most HIV replication occurs [41]–[43]. In turn, these cells might then play a role in the creation and/or maintenance of an environment in lymphoid tissues and in the thymus which would favour HIV survival and persistence by impairing protective immune responses.

In conclusion, our data support an immunopathogenic model in which the effect of HIV upon the thymus occurs through at least three mechanisms: (i) direct viral lysis which induces T-cell depletion with a preferential effect on CD4+-expressing thymocytes, (ii) bystander activation which involves all thymopoietic stages, resulting in high turnover, higher susceptibility to viral infection and exhaustion of regenerative capacity, (iii) enrichment of Treg in the thymus possibly leading to the suppression of pathogen-specific immunity and contributing to lack of control of HIV.

The fact that we found active thymopoiesis in thymic tissues isolated from HIV-infected patients despite prolonged CD4 T lymphopenia, supports the need for further investigation into strategies for the manipulation of thymic function, since true thymic reconstitution would allow repair of T-cell receptor defects that have proved recalcitrant to therapeutic intervention thus far.

The key role of T-cell activation in HIV-infected thymus in causing CD4+ thymic precursors depletion strongly indicates the need for immunological strategies aimed at targeting the pool of latently infected cells that constitute the reservoir for replication competent virus. Equally important is the necessity to develop new techniques for decreasing cellular activation, thereby reducing the number of cells targetable by the virus.

Enhancing thymic function, together with suppression of immune activation and manipulation of Treg function will be essential to restoring the function and breadth of the T-cell compartment of the cellular immune system during HIV-1 infection.
